# Returning to Work After Maternity Leave: A Qualitative Study of Nurses' Experiences and Factors Influencing Their Retention

**DOI:** 10.1002/nop2.70654

**Published:** 2026-06-21

**Authors:** Elizabeth Johnson, Jennifer Kosiol, Elizabeth Elder

**Affiliations:** ^1^ Health Services Management Griffith University Gold Coast Queensland Australia; ^2^ Centre for Applied Health Economics Griffith University Gold Coast Queensland Australia; ^3^ Menzies Health Institute Queensland Griffith University Gold Coast Queensland Australia; ^4^ School of Nursing and Midwifery Griffith University Gold Coast Queensland Australia; ^5^ Centre for Work, Organisation, and Wellbeing Griffith University Gold Coast Queensland Australia

**Keywords:** healthcare management, maternity leave, nurse management, nursing retention, return to work, workforce

## Abstract

**Aims:**

The aim of this research was to explore the experiences of nurses returning to work following maternity leave and the factors that influence them to stay or leave their positions.

**Design:**

Descriptive qualitative study.

**Methods:**

Twelve in‐depth semi‐structured interviews were conducted with nurses working within a health service in South‐East Queensland. Data were analysed thematically following the method described by Braun and Clarke. The findings were interpreted through the lens of Conservation of Resources theory.

**Results:**

Three key themes were identified: work‐family integration, support systems and organisational factors. Flexibility, supportive and emotionally intelligent managers, and positive workplace culture influenced the return‐to‐work experience and positively influenced nursing retention. Rigid rostering schedules, policy‐practice gap, poor managerial support and poor work‐life balance negatively affected the return‐to‐work experience and intention to leave.

**Conclusion:**

The study findings offer a unique contribution to existing knowledge by detailing the experiences of nurses returning to work after maternity leave. The findings of this study indicate the return‐to‐work experience is a complex, multifaceted phenomenon with many factors influencing nurses' retention.

**Implications for Profession:**

This study highlights the importance of supportive and emotionally intelligent managers, effective organisational policy and flexibility in working. Fostering positive organisational cultures that support mothers returning from maternity leave is essential in reducing work‐family conflict and ensuring a sustainable nursing workforce for the future.

**Impact:**

This research has implications for healthcare managers, organisations and policymakers. The findings emphasise the need for flexibility, improved support and strategies to improve nursing retention following maternity leave.

**Reporting Method:**

COREQ checklist was used for reporting in this study.

**Patient or Public Contribution:**

This study did not include patient or public involvement in its design, conduct or reporting.

## 1 Introduction

Returning to work following maternity leave is widely recognised as a complex and emotional transition, shaped by competing demands, identity shifts, work‐family conflict and perceived organisational support (Costantini et al. 2022; Rapisarda et al. 2024). Despite these well‐established challenges, there is limited research examining how nurses' return‐to‐work experiences following maternity leave influence retention. This is evident within a recent review highlighting the challenges nurses face surrounding shift‐work, supervisor support, lactation policy, work‐family conflict, childcare and engagement when returning to work (Zandian et al. 2020).

The concept of retention can be described as ‘a systematic attempt by an organisation to develop and nurture an encouraging environment that promotes employees' decisions to remain in the organisation’ (Coetzee et al. 2018). While returning to work following maternity leave is not explicitly identified as a key determinant of retention in existing literature (Chamanga et al. 2020; McIntyre et al. 2024; Pressley and Garside 2023), work‐family conflict has been discussed under the broader category of ‘work‐life balance’ as a key concept affecting retention. This suggests that the specific influence of maternity leave transitions on retention may be underexplored. Given the World Health Organisation's State of the World's Nursing Report 2025 (World Health Organization 2025), highlighting a global shortage of nurses, and projections that Australia will face an estimated shortfall of approximately 79,000 nurses by 2035, factors influencing nursing retention remain a critical research priority (Australian Government Department of Health and Aged Care 2024) both nationally and internationally.

Work‐family conflict, which occurs when one's family life negatively impacts their work life, has been identified as a significant factor influencing nursing retention (Zandian et al. 2020). Evidence from other professions suggests that returning to work following maternity leave can exacerbate work‐family conflict, subsequently influencing engagement and retention (Costantini et al. 2020). Women also face a range of internal and external challenges during this transition, including shifting identities, competing demands and navigating organisational support (Le Sueur and Boulton 2021). Recent qualitative research further highlights the complexity of the return‐to‐work transition, with nurses describing experiences of moral distress, role strain and inadequate organisational support, contributing to burnout and attrition (Watson, Tapp, et al. 2026; Watson, Peterson, et al. 2026).

Given the high percentage of females within the nursing profession, a significant portion of the nursing workforce is responsible for balancing work commitments alongside family, parenting and partner responsibilities (Yildiz et al. 2021). Despite the nursing workforce in Australia being overwhelmingly made up of females (Australian Government Department of Health and Aged Care 2024), there is a dearth of literature surrounding the experiences of nurses returning from maternity leave and how this impacts retention, specifically within an Australian context. The available literature suggests there are consistent challenges faced by nurses when returning to work following maternity leave including a lack of flexibility in scheduling, poor lactation support despite lactation policies, childcare issues and decreased workplace engagement (Hearfield et al. 2022; Hill et al. 2023; Tseng et al. 2023). Furthermore, nurses have described experiencing heightened stress during this period (Hill et al. 2023), and report higher levels of work‐family conflict compared with other healthcare professionals (Ekici et al. 2020).

There remains limited research exploring how these challenges shape nurses' retention following maternity leave, particularly qualitative studies within the Australian context. This is important as qualitative approaches enable in‐depth exploration of complex, subjective experiences and factors shaping them, which may not be fully captured through quantitative methods. In Australia, eligible employees can access up to 18 weeks of government funded paid parental leave, alongside employer‐based parental leave and the right to request flexible working arrangements upon returning to work (Australian Government 2024). This is significant given 58% of the nursing workforce in Australia is currently represented by females of childbearing age (Australian Government Department of Health and Aged Care 2024), highlighting the need for research in this area to help strengthen the future nursing workforce, inform policy and decision‐makers and create new strategies to support contemporary workforce needs.

## 2 The Study

### 2.1 Aim

This qualitative study explored the experiences of nurses returning to work from maternity leave and the key factors influencing their retention. The research question that underpinned this study was: ‘What are the experiences of nurses returning to work from maternity leave and the factors affecting their retention?’

## 3 Methods

### 3.1 Design

This study used a descriptive qualitative design given its ability to explore and interpret participant's views, experiences and perspectives which aligned with the research aim (Imran and Almusharraf 2023). This approach was selected to capture a range of experiences and factors influencing retention within a real world organisational context. The study was informed by an interpretivist paradigm, which assumes reality is socially constructed and best understood through participant's lived experiences (Pope and Mays 2020). This reflects the naturalistic tradition of descriptive qualitative research, in which the aim is to explore and describe experiences as reported by participants. The study was further guided by the Conservation of Resources theory (COR theory), which provided a theoretical lens for understanding how changes in personal and professional resources influence the return‐to‐work experience following maternity leave and decisions surrounding retention.

### 3.2 Theoretical Framework

This research draws on the theoretical perspective of Conservation of Resources theory (COR theory), a motivational and stress theory which posits that individuals aim to obtain and preserve resources (Hobfoll et al. 2018). Resources may include things of importance to the individual such as employment, family, health and wellbeing. When these resources are lost or threatened, individuals are likely to experience stress (Hobfoll et al. 2018). COR theory is relevant to understanding the return‐to‐work experience following maternity leave, which is often marked by shifts in personal and professional demands. COR theory provides a useful lens for interpreting how changes to resources can influence stress, the return‐to‐work experience and decisions surrounding retention.

### 3.3 Study Setting and Recruitment

The study was conducted within a large health service in southeast Queensland, Australia. The health service employs several thousand nurses across multiple hospitals, satellite facilities and community services.

Purposive sampling, snowballing (Campbell et al. 2020) and maximum variation technique (Shaheen and Pradhan 2019) were used to ensure a diverse range of perspectives and experiences were obtained. An email containing the study information was distributed to all nursing staff across the health service by the executive nursing team, which included an overview of the study, inclusion criteria and research team contact details. Interested staff were asked to contact the research team via email. Potential participants were then screened for eligibility and maximum variation. The study aimed to achieve maximum variation by including participants from a variety of work units, specialties, sites and nursing grades, which assisted in identifying common themes across a diverse range of participants (Shaheen and Pradhan 2019). Participation was voluntary, with participants free to withdraw at any time throughout.

### 3.4 Inclusion and/or Exclusion Criteria

Nursing staff working in the health service who had given birth, taken maternity leave within the past 5 years and held a position within the organisation prior to maternity leave were invited to participate. Participants who joined the organisation following maternity leave were excluded to ensure participants could reflect on their experiences before and after maternity leave within the same organisation. Promotion or temporary higher duties during or after maternity leave were not considered in participant selection and did not influence eligibility. The 5‐year period was selected given the organisation's policy allowing for temporary reductions in hours following maternity leave until the child is of school age (approximately 5 years). Selection of participants was guided by inclusion and exclusion criteria (Table 1). The study was limited to mothers as current evidence reflects different levels of work‐family conflict and experiences when compared to fathers (Aarntzen et al. 2023; Young et al. 2022).

**TABLE 1:** Inclusion and exclusion criteria.

Inclusion criteria	Exclusion criteria
Registered nurses Individuals who have given birth Episode of maternity leave within the past 5 years Existing position (full‐time, part‐time or casual) within the organisation prior to maternity leave Anyone who has remained in their existing position prior to maternity leave, resigned or returned on reduced hours	Nurses who are not registered with Australia Health Practitioner Regulation Agency Those who returned from maternity leave over 5 years ago Did not hold a position within the organisation prior to maternity leave

### 3.5 Data Collection

Data were collected via in‐depth semi‐structured interviews, between February and March 2024. All interviews were conducted by the first author (E.J.). The interview guide was informed by a comprehensive scoping review of the literature (Johnson et al. 2025) and COR theory and was developed by the first author (E.J.) with input and oversight from co‐authors J.K. and E.E. Questions focused on participants' perceptions of resource loss, gain and protection following their return‐to‐work. Example questions included: ‘What influenced your decision to stay or leave your role?’ and ‘Can you tell me about your experience when your returned to work after maternity leave?’. Prior to data collection, the interview guide was piloted, and feedback and refinements were made with the consensus of the research team.

Interviews were conducted online via Microsoft Teams, recorded with consent, and subsequently transcribed and crosschecked for accuracy. Verbal consent was obtained prior to recording each interview and was documented by the researcher in a consent log. Transcripts were returned to participants for confirmation as part of member checking. No changes were made following participant review. In accordance with institutional ethics protocols, recordings were deleted following confirmation, and only de‐identified transcripts were retained for analysis. Interviews were conducted until sufficient depth and variation in experiences were achieved and no new concepts relevant to the research aim were identified.

### 3.6 Data Analysis

Data analysis was undertaken using thematic analysis, utilising Braun and Clarke's (2006) six phase approach. Data analysis occurred concurrently with data collection, allowing emerging insights to inform subsequent interviews. An inductive approach was used to ensure that codes and themes were strongly grounded in the data. Familiarisation with the data was achieved through crosschecking the transcripts while listening to the recordings, re‐reading the transcriptions and annotating original ideas. Codes were then systematically generated and data collated under each of the relevant codes. Initial coding and analysis were conducted by the first author (E.J.), with themes developed collaboratively with co‐authors (J.K. and E.E.) through regular discussion and refinement to ensure accuracy and consistency. The themes were then reviewed, defined, and named with a clear and informative title for each. This process required ongoing reflection until the team were satisfied that the themes reflected the data collected. Initial coding generated a substantial set of codes, which were then refined and organised into categories using a coding tree. The coding tree provided a transparent framework for consolidating codes into the final theme. Once themes were established, they were examined through the lens of COR theory to interpret how participants experienced resource loss, gain and protection in relation to their return‐to‐work experience. This explicit use of theory at the interpretive stage is consistent with descriptive qualitative research where frameworks are commonly applied to enrich understanding of inductively generated findings. The final step, as described by Braun and Clarke (2006) was the generation of this manuscript.

### 3.7 Ethical Considerations

Ethical approval was obtained from the university's and relevant health service's human research ethical committee. Verbal consent was obtained from all participants prior to the interview commencing.

### 3.8 Rigour and Reflexivity

Data collection and analysis were conducted by E.J., a nurse manager with qualitative research training and a professional background in nursing management and workforce. The wider research team included two PhD‐qualified and experienced qualitative researchers who provided regular oversight and peer debriefing to enhance analytic consistency and rigour.

To ensure credibility, member checking was undertaken and findings regularly discussed and reviewed by the research team. Transferability was supported by rich descriptions of participant demographics, contexts and experiences. Dependability was maintained through a clearly documented coding and theme development process, enabling transparency and traceability. Confirmability was strengthened by keeping an audit trail and engaging in critical dialogue with members of the research team. Reflexivity was ongoing and involved post‐interview reflections, journaling and team discussions to identify and manage potential biases. The Consolidated Criteria for Reporting Qualitative Research (COREQ) checklist (Booth et al. 2014) guided study design and reporting to enhance trustworthiness.

## 4 Findings

### 4.1 Characteristics of Participants

The study included 12 participants, aged between 28 and 43 years, and incorporated Registered Nurses, Clinical Nurses and one Acting Nurse Unit Manager. Episodes of maternity leave per person varied between one and three, as did the number of children. Two participants had twins during their maternity leave. Most of the participants remained in the same role since maternity leave, with almost all participants working reduced hours. Participants worked across a range of specialities, including critical care, medicine, surgery and day units (outpatients/non‐clinical services). Episodes of maternity leave ranged from 12 months to 2 years (see Table 2). Participants were Registered Nurses (RNs) registered with the Australian Health Practitioner Regulation Agency. Position titles such as Clinical Nurse (CN) and Nurse Unit Manager (NUM) represent classifications within the RN scope of practice. Nurse Unit Managers are senior nursing leaders responsible for the management and oversight of operations, workforce and delivery of patient care within a unit.

**TABLE 2:** Participant demographics.

Age	Number of children	Episodes of maternity leave	Level	Same role since maternity leave	Reduced hours since returning from maternity leave
28	2	1	RN	Yes	Yes
29	2	1	RN	Yes	Yes
31	1	1	RN	Yes	Yes
33	2	2	CN	Yes	Yes
34	2	2	CN	Yes	No
35	3	3	RN	Yes	Yes
35	1	1	A/NUM	Yes	Yes
35	3	2	CN	Yes	Yes
37	2	2	CN	Yes	Yes
37	3	3	CN	No	Yes
40	3	3	CN	No	Yes
43	3	3	RN	Yes	No

Abbreviations: A/NUM, acting nurse unit manager; CN, clinical nurse; RN, registered nurse.

### 4.2 Themes

**FIGURE 1:**
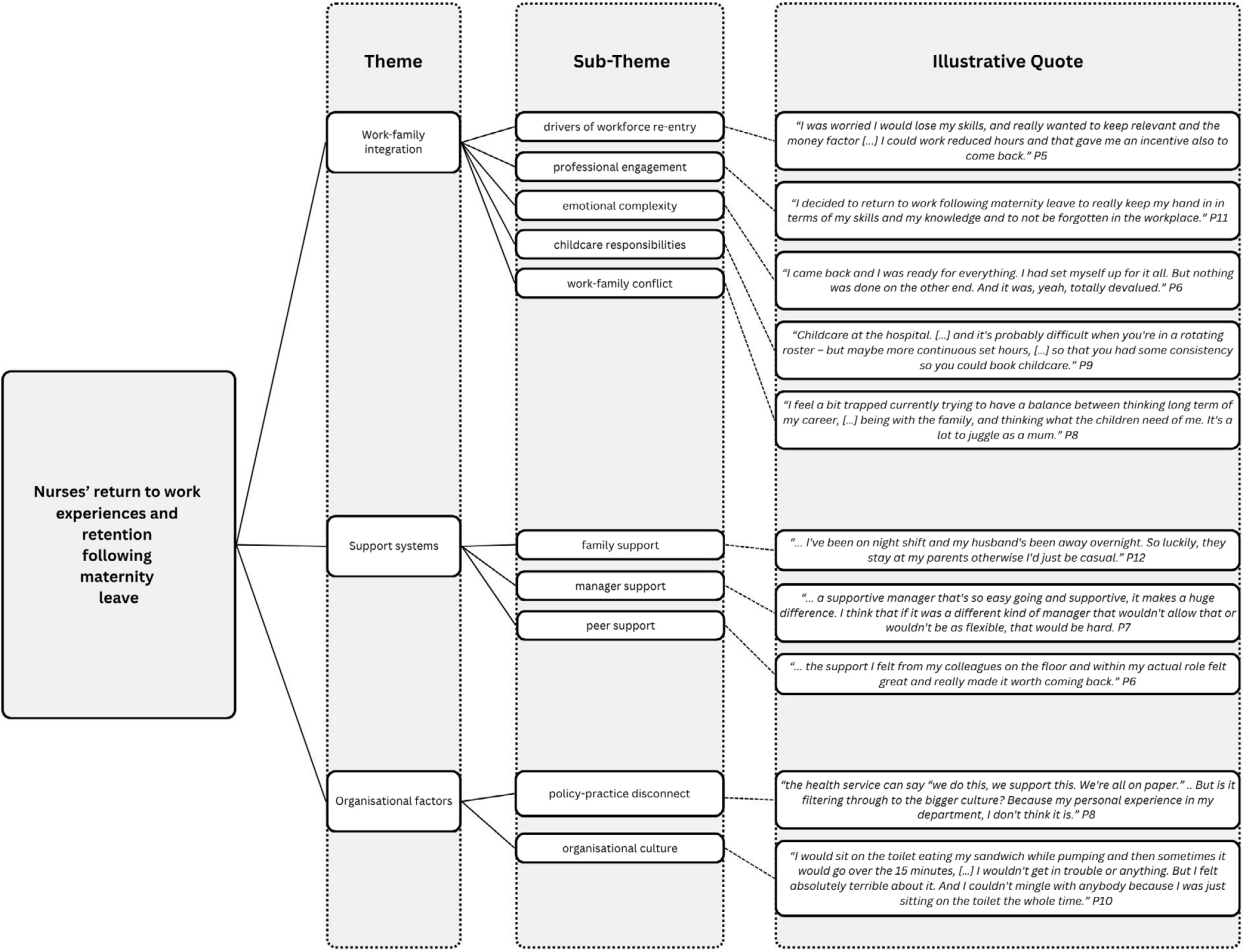
Hierarchical coding tree of themes and subthemes.

Three overarching themes were identified: (i) work‐family integration; (ii) support systems; (iii) organisational factors. These themes, along with their corresponding subthemes, address both the experiences of nurses returning to work and factors influencing their retention. The interconnected nature of these themes highlights how they collectively shape nurses' experiences and their decisions to stay or leave their roles. While each subtheme is illustrated with a representative quote for clarity and conciseness, all themes and subthemes were developed from patterns identified across multiple participants. Figure 1 outlines a hierarchical coding tree, illustrating themes, subthemes and participant quotes.

#### 4.2.1 Work‐Family Integration

Nurses' return‐to‐work experiences were shaped by their ability to balance professional and family roles. Work‐family integration emerged as a central concern, influenced by financial necessity, emotional challenges, childcare demands and work‐family conflict. These overlapping factors affected both their transition and experience returning to work and their decisions to stay or leave.

##### 4.2.1.1 Drivers of Workforce Re‐Entry After Maternity Leave

Financial necessity was a primary reason cited for returning, with many expressing a desire to remain at home for longer if not for economic pressures. Working reduced hours after maternity leave was common in an attempt to balance work and family responsibilities. As P1 explained, balancing the need to provide for their family with the desire to spend time with them was a significant challenge in their return‐to‐work experience:

Once my child turned one, I put them into daycare and then looked at basically financial wise what would work for our family. [I] was trying to find a balance between still being at home with the kids, but also, you know, being able to […] earn enough to live. (P1)

Intrinsic factors were also noted to influence the desire to return‐to‐work, with several participants emphasising the importance of re‐establishing their personal and professional identities as a driver for returning to work. For staff who expressed this, returning wasn't a necessity, but instead an opportunity to maintain their professional skills and regain a sense of self after having children:

I basically decided to return to work following maternity leave just to really keep my hand in terms of my skills and my knowledge […] And then outside of that was more just to gain that other part of my identity back post children. (P11)

##### 4.2.1.2 Professional Engagement

Participants commonly described a strong emotional connection to their professional identity, with passion and commitment to nursing motivating their return. However, many reported a diminished capacity to engage at the same intensity due to competing demands and internal conflict. This shift was both psychological and logistical, reflecting a broader struggle shared across the cohort. One participant illustrated this challenge:

It's such a change to your entire life and your whole world flips upside down when you become a parent. I was really passionate about [nursing]… But then I found it really hard to let it go during that period of maternity leave. And I felt like it would be quite difficult to enter back into the workforce with the same intensity. (P11)

The difficulty in maintaining previous levels of engagement was exacerbated when participants felt undervalued in their workplace, further eroding engagement and morale. For some, this led to feelings of detachment and questioning their professional worth:

It has made me feel very much like it doesn't matter whether I turn up or not… well, am I really valued here, or should I be looking for something else? (P4)

While professional growth remained a priority for some, most described choosing stability over progression to balance family responsibilities. Several nurses noted feeling ‘trapped’ in their roles, particularly when working reduced hours:

Moving into another position while on reduced hours would be too difficult […]. Until my reduced hours are up, I probably won't go and seek anything else. (P9)

##### 4.2.1.3 Emotional Complexity

Participants expressed a range of emotional responses to returning to work, often describing mixed feelings of anxiety, guilt and internal pressure to perform at pre‐maternity levels. The emotional strain of leaving their child, combined with the demands of re‐engaging professionally, contributed to what many described as a complex and challenging transition. This emotional complexity intertwined with broader themes such as work‐family conflict, support systems and organisational culture. One participant captured these shared sentiments:

I really lost confidence first time around […] I felt so anxious in myself returning and […] I was really dreading the return‐to‐work experience day. And to start with, I really struggled with the separation from my daughter. I would FaceTime her on my breaks […] I found the 12‐hour days really hard. Like you leave at 6:00 in the morning, get home at 8 at night. You basically don't see your child that whole day. (P12)

##### 4.2.1.4 Childcare Responsibilities

Difficulties aligning childcare with varying shift patterns, and the financial burden of childcare costs were common concerns. These issues were consistent across work units, roles and hours worked. Several participants relied on family support to meet childcare needs. When scheduled shifts did not align with set childcare days, this became a significant source of stress. One participant illustrated this common challenge:

I was rostered on days when I didn't have childcare and I would not be working days when I did have childcare. … it cost me money that it probably didn't need to if I'd had the flexibility in my shifts or if I'd been able to say ‘I'm only available these days because, this is when I've got childcare’ and actually got those shifts. (P4)

In addition to financial strain, the mismatch between work schedules and childcare availability contributed to frustration and compounded the emotional burden of balancing professional and family responsibilities. Another participant described the inflexibility in scheduling:

It definitely is a big thing, especially morning shifts. During the week I try and do late shifts. On the weekends I can work early shifts. But unless my husband takes an RDO or something, or a carer's day. I pretty much can't get to work. (P9)

These experiences were typical across the cohort and reinforced the broader themes of work‐family conflict and emotional complexity. On‐site childcare and more flexible rostering practices were commonly suggested as solutions to support working parents and reduce the strain associated with childcare.

##### 4.2.1.5 Work‐Family Conflict

Work‐family conflict negatively impacted the return‐to‐work experience. The complex interplay of shiftwork, childcare, infant feeding, and balancing parenting and professional responsibilities intensified this conflict. For example, P9 described the challenges of arranging care during irregular hours:

Who would care for him? Especially if we were working odd hours, it wasn't like we could access daycare easily or we had set hours where we could access daycare […] I was still breastfeeding when I returned to work […] how would my husband go getting him to bed when he wouldn't go down for anybody else? And I had never been away from him overnight, now I'm suddenly doing night shifts and working late and all that sort of thing […] You've got sick children, breastfeeding, pumping. But yeah, not having him in the odd hours was particularly hard. (P9)

Some participants felt trapped in their roles, needing to sacrifice or delay career goals to manage these competing demands. As P8 reflected, balancing work, family commitments and ‘what the children need of me’ generated significant stress and emotional burden, often accompanied by guilt and self‐doubt:

It's a lot to juggle as a mum. (P8)

This internalised pressure to meet professional commitments despite new caregiving responsibilities was echoed by P6 who described feelings of guilt about their reliability as an employee:

I want to be the employee that's reliable and turns up and does their job and you know, but obviously that is now different. I'm at the beck and call of my child and their daycare germs […] So my sick leave is not my own. So I faced some challenges in feeling guilty that I wasn't as reliable as I had previously been as an employee. (P6)

#### 4.2.2 Support Systems

Support systems heavily influenced nurses' experiences and contributed to retention following maternity leave. Support was classified into three main groups: (i) family support, (ii) peer support, and (iii) manager support. Strong support systems contributed to higher morale, improved return‐to‐work experiences, and increased intention to stay in their roles.

##### 4.2.2.1 Family Support

Family support was deemed crucial and enabled nurses to increase their shift availability, maintain permanent employment and reduce work‐family conflict and stress. Due to the mismatch of childcare operating hours and nursing shift patterns, participants identified the need to rely on partners or family to take their children to and from care, demonstrating the pivotal role of family in managing work demands:

I'm lucky my husband works a nine to five sort of job, and so he can do pickups and drop offs at appropriate times because the other part of that is that most childcare centres don't open till 6:30 and when you start work at 7:00, that is not a do‐able drop off when you live more than 10 minutes from the hospital. (P4)

##### 4.2.2.2 Peer Support

Peer support took the form of knowledge sharing by helping one another navigate the return‐to‐work experience, policies and processes, as well as providing empowerment and emotional support in their motherhood and working roles. Peer support was predominantly informal and positively influenced the return‐to‐work experience:

The people I work with for the most part, are amazing and most of us are in the same sort of boat or getting close to being in the same sort of boat. So, we're all very supportive of each other and empowering of each other to do what we need to do for our families. (P4)

Peer support was often reciprocal, with participants offering empathy and care to others based on their own experiences. This connection and peer support emerged as a key factor in enhancing the return‐to‐work experience:

I'm now even more in tune. Like when there are mums coming back to the workforce, I'm like, ‘are you OK? How's your first shift?’ […] I think if you haven't walked in their shoes, you don't know how it might be feeling. (P12)

##### 4.2.2.3 Manager Support

Perceived managerial support varied across the study and was highlighted as crucial in fostering a positive return‐to‐work experience and significantly influenced retention. Having a supportive manager was associated with a positive return‐to‐work experience. Managers who had children themselves were considered ‘game changers’ (P5) as they often demonstrated greater understanding and empathy towards nurses returning to work from maternity leave:

To have a supportive manager that's so easy going and supportive, it makes a huge difference. I think that if it was a different kind of manager that wouldn't allow that or you know wouldn't be as flexible, that would be hard. So that makes a huge, huge impact … (P7)

Most participants agreed that approachability, empathy, flexibility, and understanding of employee's experience—akin to emotionally intelligent managers—were key elements of managerial support and positively impacted their return‐to‐work experience. However, in contrast, not all managers possessed these qualities. Some participants suggested that, at times, they were exposed to ‘really unprofessional’ (P8) behaviours, with management openly being unsupportive of employees returning to work after maternity leave, making their re‐integration more difficult. For example:

… everyone knows, that she doesn't really like childbearing age women, and she's actually said to me in an office […] ‘oh, you know, women just change after they have a baby and come back to work’ and it's like she was referring to I don't know, their drive. (P8)

P9 discussed the emotional impact of poor managerial support on their return‐to‐work experience, citing an email that was sent only to the mothers within their unit, resulting in feelings of guilt and distress among the staff:

Recently an e‐mail went out to the mums. There's five of us out of about 75 staff. I think the intentions were probably better than it was perceived. […] I think it sort of came across as ‘don't expect to get what you want.’ I know we have one girl who's just come back, she was in tears over it. Another one who feels like she's too guilty to be there. (P9)

For many participants, managerial support in providing flexibility in work schedules was vital in contributing to a positive return‐to‐work experience. Being allowed to swap shifts and having an accommodating roster positively influenced the return‐to‐work experience. These strategies also helped reduce work‐family conflict. For example:

We have an amazing NUM. He's so supportive. And I mean only working one day a week, they're OK with that. And he's really accommodating. I've said like ‘hey, I prefer to work earlier than that. That way I can come home at a decent time.’ So he put me down on 730 starts since I've come back. And that's amazing.[…] I can still put him to bed and still do that nighttime routine. And doing that one day a week is awesome. (P7)

In contrast, some managers compared those returning to work on reduced hours to their fulltime staff, ‘oh, well, our full timers do this …’ (P9), leading to some feeling devalued and dehumanised in the workplace:

It makes you feel like a number. Like your employee number. You try your best and then you still feel like you're an annoyance to them. (P9)

#### 4.2.3 Organisational Factors

Organisational factors, particularly policy‐practice disconnects and workplace culture, emerged as key determinants of nurses' return‐to‐work experiences and retention following maternity leave. Across the dataset, participants described a consistent gap between the organisation's supportive policies (e.g., flexible work arrangements, lactation breaks and job‐sharing) and the inconsistent or obstructive ways these were implemented in practice. These structural and cultural challenges, often rooted in specific unit‐level norms, contributed to diminished morale, compromised professional identity and increased intention to leave.

##### 4.2.3.1 Policy‐Practice Disconnect

Many participants reported that, despite formal policies supporting work‐life balance, in practice, these were poorly implemented or actively discouraged. There was a recurring sense that using entitlements, such as flexible hours or lactation breaks, required self‐advocacy and often triggered guilt or pushback. As one participant shared:

Organisationally there's all these things in place that are great. I just don't know if they come to fruition […] or feel like you can utilise them. All these return‐to‐work experience agreements and procedures […] I've had to show people the policies on it, even the mothers who have been feeling guilty […]you're allowed to do this – you're allowed to be on reduced hours, you're allowed to make requests, and even things like breastfeed, pumping breaks. (P9)

When rostering was inflexible, some staff were indirectly encouraged to call in sick to manage family responsibilities. This was an institutional workaround that participants found troubling:

I think one of the things that [the organisation] would never like to hear is that people are calling in sick because they can't make their shifts work … Again, [they] rostered me on to a day that I cannot work, and I turned up to work and [they] go ‘we don't normally see you on a Monday.’ I said ‘You rostered it. But no, I don't normally work Mondays because I don't have childcare.’ And the response, was ‘I expected you to call in sick.’ (P4)

A gap between organisational policy and practical implementation was evident. While supportive policies and procedures, such as flexible working arrangements (FWAs), reduced hours, and lactation breaks are in place, they are not always fully understood, utilised or encouraged, resulting in feelings of guilt and frustration. Limitations in flexibility and support left participants questioning the reality of family‐friendly practices:

And I think the other thing is [the organisation] say this ‘oh, we're family friendly’ but putting in restrictions about what people can roster or not roster permanently and not really allowing the flexibility for families, actually doesn't make them appear that way. (P4)

FWAs are designed to assist staff in achieving work‐life balance, with participants commonly requesting them to support family and childcare needs. While supported at an organisational level, several participants expressed challenges surrounding FWA processes. This resulted in participants feeling devalued and demoralised as a result:

… that was a very challenging process […] and lots of barriers put in place where I felt like the objective was ‘just keep putting barriers in her place and she'll eventually either leave’ – which was explicitly offered to me – or she'll comply with being more available. It definitely makes you feel like you're a number. And that prior performance, or prior skills and knowledge are not valued – you're just replaceable […] the implication was definitely that I was replaceable very easily. (P11)

Many participants highlighted challenges around breastfeeding due to inadequate facilities, and poor privacy despite organisational policies supporting breastfeeding and lactation breaks. A lack of facilities meant that some staff ‘would sit on the toilet eating my sandwich while pumping’ (P10). Experiences like these led feelings of guilt, and as though they were letting their team down. As a result, some chose to end their breastfeeding journey early:

I didn't have anyone that could cover me, and I also felt within myself that I was letting the team down by being off the floor […] The second time I cut it off straight away. Pretty much I was like, ‘oh, we're not doing this anymore’. […] And then it sort of just faded. (P2)

These disconnects between policy and practice were associated with a diminished sense of belonging, professional worth and confidence in organisational support for returning mothers.

##### 4.2.3.2 Organisational Culture

Cultural norms within some work units reflected implicit biases against mothers, reinforcing feelings of marginalisation. Participants described an ‘undercurrent’ suggesting that prioritising family life meant reduced professional commitment:

… There's definitely that unspoken undercurrent in the workplace, where it's like, oh, ‘OK, well, you're a mum now.’ (P11)

Some reported being deliberately targeted or undermined due to their parental responsibilities, referring to it as ‘the mum thing’:

The comment got back to me that it was ‘well, she hasn't requested her night shift, so I'm going to give her one here because I know she can't do it and it'll make it really inconvenient […] And I thought, why would you do that to me? Why be so vindictive? … I think it's the mum thing.’ (P9)

Some feared if work were aware of their pregnancy status, they would be overlooked for promotional opportunities:

When I went for my [x] interview when I was pregnant with my first child. I had really severe hyperemesis the entire time with both kids. But I knew that if they knew I was pregnant, that I probably wouldn't get the job. So, I vomited before the interview and vomited after the interview. (P9)

A critical way organisational culture influenced retention was through limiting career progression opportunities for returning mothers, often due to rigid expectations regarding work hours and availability:

… I thought I could start doing the NUM career progression and then have kids […] In which [the NUM] was like, well, actually, no, you're probably better off being a CN and returning as a CN to have flexibility in the shifts, because if you return as a NUM you have to work full time and it's really difficult to job share. (P3)

Inadequate scheduling flexibility to accommodate set childcare days exemplified the organisational rigidity, further reinforcing work‐family conflict. P11 illustrated how increasing hours was only acceptable if they were to increase their late and night shifts which conflicted with their family responsibilities. In some cases, this rigidity influenced the number of hours mothers would return‐to‐work at:

All I really needed was not just the understanding that I can only work certain days due to childcare in that Monday to Friday period, but the night shifts and the really late shifts […] – really limiting them. […] And there was no allowance if I wanted to increase my hours, it would also mean agreeing to increase the amount of night shifts, […], and late shifts that I would have to do. Which I just couldn't support on a personal level with kids in the house and a husband who worked long hours and often away. (P11)

Cultural norms appeared to be isolated to specific units and were not reflected across the wider organisation with participants hopeful it may be different within other units. This was affirmed by others who described elements of positive workplace cultures and return‐to‐work experiences. This was evident in P7s love for their role, unit, and the sense of trust and engagement which positively influenced their experience:

…work hours, the team that we have there, with amazing patients that we see day‐to‐day are…amazing. You know everything. It's just it's a really good place to work. Really. Like they're fair, everyone's helpful, approachable…. (P7)

Overall, organisational factors, particularly inconsistency in policy application and unsupportive cultural norms, played a central role in shaping the return‐to‐work experience and nurses' long‐term career decisions. These structural barriers often intersected with personal resource loss, as conceptualised within COR theory, influencing participants' retention and professional trajectories. Table 3 provides a summary of this interpretation.

**TABLE 3:** Themes interpreted through the lens of COR theory.

Theme	COR concept	How resources are lost/gained	Influence on retention
Work‐family integration	Resource loss and resource threat	Competing demands, emotional strain, childcare challenges, loss of balance between work and self.	Resource loss spirals increase stress and result in increased intention to leave.
Support systems	Resource gain, resource loss and resource threat, resource caravan passageways	Emotional, practical, and information support from peers, family and managers increase resources and enhance the return‐to‐work experience. When support is absent, resource loss occurs.	Resource gain cycles offer increased confidence and security, supporting retention. Resource loss spirals increase stress and intention to leave.
Organisational factors	Resource loss and lack of resource gain, disrupted resource caravan passageways	Policy‐practice disconnect, poor policy implementation, rigid scheduling, decreased career progression opportunities lead to resource loss and a lack of resource gain.	Ongoing resource loss and ineffective resource caravan passageways lead to dissatisfaction, feeling devalued, and greater intention to leave.

## 5 Discussion

The aim of this qualitative study was to understand the nurses' return‐to‐work experience following maternity leave and the factors influencing retention. The research addressed the question: ‘What are the experiences of nurses returning from maternity leave and the factors that influence their retention?’. This was achieved through conducting semi‐structured interviews with 12 participants within a large health service in Queensland, Australia. The findings of this study offer new perspectives within the Australian healthcare setting, during a time of changing workforce needs and critical workforce shortages.

Nurses described varied but interrelated experiences, shaped by work‐family integration, support systems and organisational factors. These findings suggest that retention is influenced by individual circumstances, as well as the interaction between organisational conditions and available support.

### 5.1 Challenges Integrating Work and Family Life

Work‐family integration was a central feature of participants' return‐to‐work experiences and was shaped by competing demands, identity shifts and organisational context. Drivers of workforce re‐entry included financial reasons and re‐gaining identity after having children, which resonated across existing literature (Costantini et al. 2022; Hill et al. 2023). While participants were motivated to return to work, some felt unable to engage with the same level of intensity as pre‐maternity leave. However, this reduced engagement appears to be only temporary and influenced by organisational support and management practices (Hill et al. 2023). Participants experienced multiple and often conflicting emotions, alongside internalised pressure to perform at pre‐maternity levels, resulting in feelings of anxiety and separation from their children. This is consistent with literature describing the return‐to‐work experience as challenging, emotionally charged and complicated by internal conflict (Costantini et al. 2022; Rapisarda et al. 2024). Re‐orientation programmes, gradual return‐to‐work processes, flexibility to support work‐life balance and maternity coaching models have been demonstrated as effective means to support mothers emotionally when returning to work, while also increasing engagement (Hill et al. 2023). Emerging evidence continues to highlight the complexity of the return‐to‐work experience and the need for structured organisational support (Watson, Peterson, et al. 2026). However, these appear to be unexplored within the nursing context and warrant further investigation.

Childcare was a significant stressor, with participants struggling to align fixed childcare days with rotating schedules, meaning they were often rostered to work shifts where they didn't have childcare and vice versa. This contributed to financial strain and increased work‐family conflict, often requiring alternative care arrangements for their child or utilising sick leave.

These findings are consistent with existing literature highlighting access to childcare and reliance on family support as key challenges and barriers (Tseng et al. 2023; Hobfoll et al. 2018; Riaz and Condon 2019). However, reliance on family is not always a realistic solution, as family may not be able to assist. On‐site childcare was suggested as a solution by participants, which has been demonstrated to improve recruitment and retention of healthcare workers (Braddock et al. 2023). Internationally, on‐site childcare is gaining government attention and was recently recommended in the National Health Service Long Term Workforce Plan as a means of sustaining the workforce (National Health Service 2023).

Participants illustrated the need for balance between their work and family life, which often led to increased work‐family conflict. This demonstrated the complex interplay of factors, as it was interrelated and dependent on support systems, childcare and perceived emotional intelligence and understanding of nurse managers. Nurses experience higher levels of work‐family conflict when compared to other healthcare professionals, likely due to irregular hours and rotating shifts (Ekici et al. 2020). In a study involving 378 nurses across six hospitals in Iran, 93% of participants expressed moderate to high levels of work‐family conflict which impacted their quality of work and home life (Zandian et al. 2020). This is important given that work‐family conflict has been identified as a key factor influencing nurses' organisational commitment (Fukuzaki et al. 2021) and intention to leave (Yildiz et al. 2021; Fei et al. 2023; Pennbrant and Dåderman 2021; Phuekphan et al. 2021).

Taken together, these findings suggest that work‐family integration is not shaped solely by individual factors but is also influenced by organisational structures, flexibility and access to meaningful support.

### 5.2 Support System Influences

Family support was integral in enabling nurses to increase work hours, alleviating childcare concerns and mitigating work‐family conflict. Conversely, the absence of family support was a factor noted to influence intention to leave which is consistent with the broader literature (Costantini et al. 2022). Peer support also positively influenced return‐to‐work experiences and intention to stay, with nurses relying on peers for knowledge sharing and guidance in navigating return‐to‐work processes. Peer support also occurred on a deeper level, with nurses supporting and empathising with one another through similar experiences and helping one another navigate cultural norms within individual units. Social support from peers has been identified as a key factor positively influencing nursing retention (Costantini et al. 2022; McIntyre et al. 2024). While not directly measured, these findings suggest peer support may play an important role in supporting nursing retention following return from maternity leave.

Managerial support varied within this study, with some citing positive experiences of supportive managers who demonstrated empathy, understanding and flexibility in scheduling, positively influencing the return‐to‐work experience and intention to stay. In contrast, others faced challenges with a perceived lack of support, little understanding and poor flexibility in schedules which negatively impacted their return‐to‐work experience. In some cases, this resulted in participants feeling devalued and negatively impacted intention to stay. These findings highlight the importance of managers having an awareness of the complex emotional challenges nurses face when returning to work from maternity leave. This supports existing literature suggesting family‐supportive supervisor behaviour is a key factor in workplace thriving, helping staff achieve work‐life balance (Griffin et al. 2023; Guo et al. 2024), and supporting retention (Yamamoto et al. 2024). These behaviours are defined as those that support families in four key areas: emotional support, instrumental support, role modelling behaviours and work‐family management. This results in enhanced support and flexibility, assisting the employee in managing professional and family responsibilities (Zhang et al. 2020).

### 5.3 Organisational Influences

Organisational factors encompassed policy‐practice disconnect and workplace culture. In this study, policy‐practice disconnect described the perceived disconnect between organisational intent and practice. Some participants reported positive experiences in navigating FWAs and being supported in temporarily reducing contracted work hours. In contrast, several participants discussed feeling supported at an organisational level due to supportive policies yet had failed to see these translate to practice or had faced significant challenges in their requests for these.

Lactation in the workplace was a common challenge discussed by participants within this study. Despite policy supporting lactation breaks, participants experienced barriers utilising these due to internal guilt about letting their team down or needing to pump over the allocated timeframe. Furthermore, due to inadequate facilities or these being located too far from their work unit, participants were often forced to use unsuitable areas such as offices, unlockable shared rooms, areas accessible by patients and toilets. These barriers contributed to some nurses ceasing breastfeeding earlier than intended. This supports existing evidence where nurses have cited expressing in storerooms, toilets, and offices, and interruptions while expressing despite organisational lactation policies (Hearfield et al. 2022; Hill et al. 2023; Riaz and Condon 2019) These findings demonstrate an important gap between policy and practice, with direct impact on both wellbeing and workforce participation.

Challenges surrounding FWA's were particularly prominent, resulting in many participants feeling devalued, replaceable, and dehumanised in the workplace due to being ‘treated as a number’. This reinforces the gap between organisational intent and managerial practice, suggesting that policy alone is insufficient without consistent and supportive implementation. The findings within this study confirm existing evidence in the healthcare workforce demonstrating a lack of standardised implementation of FWA's and variation in policy application between managers and individual units (Hulcombe et al. 2021).

Overall, policy‐practice disconnect undermines perceived organisational support, contributes to feelings of being devalued and negatively influences intention to stay as highlighted in this study. Despite its impact, this disconnect remains underexplored in the health workforce context and warrants further investigation.

Organisational culture is a key factor influencing nursing retention within organisations (Marufu et al. 2021) and was highlighted within this study as shaping both retention and return‐to‐work experiences. Positive workplace culture supported job embeddedness and retention following maternity leave, whereas unsupportive cultural norms or attitudes towards mothers contribute to feeling devalued and elements of workplace dehumanisation.

While a relatively emerging concept in health workforce literature, dehumanisation and devaluation are negatively associated with retention, staff engagement and intention to stay (Baldissarri and Fourie 2023). These findings reinforce the importance of supportive workplace cultures, effective policy implementation and family‐supportive supervisor behaviours in promoting positive experiences and retention following maternity leave.

### 5.4 Workforce Retention Strategies

Decisions to stay or leave following maternity leave were shaped by work‐family conflict, support structures, team connection, flexibility and job embeddedness. While many participants sought career progression, balancing ambitions with work and family demands often led them to prioritise comfort, stability and flexibility. Many participants described an intent to leave their current roles, mostly in pursuit of career advancement or roles offering greater flexibility or improved work‐family balance. Importantly, this intention to leave was typically directed at specific roles rather than the organisation, with participants expressing a preference for internal opportunities demonstrating organisational commitment. These findings extend existing literature by highlighting how organisational conditions shape the return‐to‐work experience and longer term perceptions of career progression and professional value.

Findings from this study suggest that working reduced hours after maternity leave may influence career progression within the organisation. Participants described both structural constraints such as being unable to work full‐time and cultural norms as limiting career progression while caring for young children. These findings also suggest that returning to work following maternity leave may involve re‐negotiating one's professional identity. This is consistent with literature describing professional identity as dynamic and shaped by role, work environment and recognition (Kaiser et al. 2026) This may contribute to nurses feeling disenfranchised by the organisation, particularly given that they return motivated and engaged yet perceive their opportunities to grow as reduced.

While the organisation supports job‐share and flexible working arrangements, this did not always appear attainable for participants. This is despite participants identifying these arrangements as key enablers supporting flexibility, career progression and retention, which is consistent with existing literature (Pressley and Garside 2023; Dousin et al. 2021). This highlights an example of policy‐practice disconnect and raises important considerations regarding potential inequities in workplace practices, which may inadvertently disadvantage employees with caregiving responsibilities. These findings may also have implications for perceived discrimination in the workplace.

These challenges are further complicated by the current global workforce crisis, as well as changing generational needs and attitudes of the workforce (Kim et al. 2024). Strategies aimed at improving employee engagement and work‐family balance should be prioritised by organisations as an instrumental means of supporting nurses and assisting in retention following the return‐to‐work period. Evidence suggests organisational support, flexibility in scheduling and easier access to FWA's are critical in improving work‐family balance and decreasing intention to leave and therefore should be considered as key priorities (Kim et al. 2024).

### 5.5 Theoretical Implications

The findings of this study can be interpreted through the lens of COR theory. Within this study, resources such as work‐family balance, managerial support, flexibility and career opportunities were identified as critical in shaping the return‐to‐work experience. Nurses who gained these resources reported reduced stress and increased intention to stay, while those who experienced resource loss described heightened conflict, dissatisfaction and intention to leave. From an organisational perspective, resource caravan passageways such as policy implementation, policy‐practice disconnect and support were critical in gaining or losing resources. This application of COR theory builds on existing literature that has used COR to explore work‐family conflict and retention among nurses (Pennbrant and Dåderman 2021), adding new insights by focusing specifically on the post‐maternity leave period in the Australian Healthcare context. These findings suggest COR is a useful framework for understanding nursing workforce retention, highlighting the importance of resource preservation during career transitions such as maternity leave.

### 5.6 Strengths and Limitations of the Work

The findings generated from this study lay the foundation for future research, with recommendations to improve practice. This research contributes in terms of understanding the experience and factors influencing nursing retention following maternity leave, a contemporary understanding of nursing workforce needs, and how to best support nurses and assist in retention following maternity leave.

There were several limitations to this study. First, no participants external to the organisation were recruited, meaning that nurses who may have resigned following maternity leave were not captured, which would provide a more comprehensive understanding. Management perspectives were also not included in this study, which could provide valuable insights into the challenges they encounter implementing family‐supportive practices and organisational policies. While the findings from this study provide a solid foundation for understanding the nurses' experiences returning from maternity leave, they are based off a single health service context.

### 5.7 Recommendations for Further Research

Areas of future research should consider widening the scope of this study to include other health services, participants who have already resigned, and incorporating the perspectives of managers and healthcare leaders. Exploring and developing maternity coaching models within a nursing context would be beneficial given its evidence of success in other industries. This could include the development of a coaching model that captures nurses prior to the commencement of their maternity leave, as well as following their return to work and beyond. Exploring the perspective of fathers/males as the primary carer should also be considered. Further research to understand the full experience of job‐sharing in the nursing context would be beneficial. Finally, future research should also examine the policy‐practice disconnect where formal policies exist but are not consistently enacted in practice, which would provide better understanding of how this gap influences wellbeing, return‐to‐work experiences, organisational culture and retention.

### 5.8 Implications for Policy and Practice

Policies should be reviewed to identify gaps or disconnects, and that they support contemporary workforce needs. A working group consisting of stakeholders from the early childhood industry, state and local governments, hospital management, and staff with lived experience could improve childcare access. On‐site childcare should be considered given it has been identified as a factor that helps to support nurse retention (Braddock et al. 2023).

For practice, training for managers regarding policy implementation, emotional intelligence and family‐supportive supervisor behaviours would contribute to greater support, consistency and reduction of policy‐practice disconnect. Strategies that support improved work‐family balance such as greater access to job‐share opportunities should also be considered to support flexibility and career progression for nurses (Pressley and Garside 2023; Dousin et al. 2021). Implementation of maternity‐leave coaching models should also be considered given their evidence in supporting emotional, physical and practical support of working mothers (Le Sueur and Boulton 2021; Dzingwa and Terblanche 2024; Moffett 2018). The development and implementation of a maternity leave coaching model contextualised to nursing warrants consideration.

## 6 Conclusion

The findings of this explorative study offer a unique contribution to existing knowledge by providing evidence detailing the experiences of nurses returning to work after maternity leave. These findings clearly indicate returning to work following maternity leave is a complex, multifaceted phenomenon with many factors influencing nurses' retention. work‐family integration, support systems and organisational factors were identified as key factors influencing the return‐to‐work experience and retention following maternity leave.

Ultimately, flexibility, supportive and emotionally intelligent managers and positive workplace cultures influenced the return‐to‐work experience and intention to stay. It was also identified that some nurses stayed within their roles following maternity leave due to feeling ‘trapped’. Conversely, rigid scheduling, organisational factors, poorly executed policy, lack of managerial support and poor work‐family balance significantly impacted the return‐to‐work experience while also influencing intention to leave. The pursuit of improved work‐family balance was also identified as a key reason influencing intention to leave. These findings also highlight the importance of organisational practices in shaping nurses' perceptions of professional value and career progression following maternity leave.

## Author Contributions

All the authors made substantial contributions to conception and design, or acquisition of data, or analysis and interpretation of data; involved in drafting the manuscript or revising it critically for important intellectual content; given final approval of the version to be published. Each author have participated sufficiently in the work to take public responsibility for appropriate portions of the content; agreed to be accountable for all aspects of the work in ensuring that questions related to the accuracy or integrity of any part of the work are appropriately investigated and resolved.

## Funding

The authors have nothing to report.

## Ethics Statement

Ethics approval was obtained from the Griffith University and the relevant health service's human research ethical committee on 13th December, 2023 (HREC/2023/QGC/104023). The identity of the HREC has been anonymised in the manuscript for confidentiality and to protect the health service.

## Conflicts of Interest

The authors declare no conflicts of interest.

## Data Availability

The data that support the findings of this study are available on request from the corresponding author. The data are not publicly available due to privacy or ethical restrictions.
